# A Time Delay Predator-Prey System with Three-Stage-Structure

**DOI:** 10.1155/2014/512838

**Published:** 2014-03-27

**Authors:** Qiaoqin Gao, Zhen Jin

**Affiliations:** ^1^Department of Mathematics, Lvliang University, Lishi, Lvliang City, Shanxi 033001, China; ^2^Department of Mathematics, Shanxi University, Taiyuan, Shanxi 030006, China

## Abstract

A predator-prey system was studied that has a discrete delay, stage-structure, and Beddington-DeAngelis functional response, where predator species has three stages, immature, mature, and old age stages. By using of Mawhin's continuous theorem of coincidence degree theory, a sufficient condition is obtained for the existence of a positive periodic solution.

## 1. Introduction

The dynamic relationship between the predator and the prey has long been and will continue to be one of the dominant themes in both ecology and mathematical ecology due to its universal existence and importance. The traditional predator-prey model has been studied extensively (see [1–3] and references cited therein). Since the pioneering and original work of Aiello and Freedman (see [4]) considered the difference between immature population and mature population, stage-structured models have attracted great attention and have been extensively researched in recent years (see, e.g., [5–9] and the references cited therein). They studied the stage-structured model with time delay, cannibalism, impulsive harvesting strategies and response functions, and many interesting results concerning stability of equilibrium state and existence of periodic solutions. At present, many authors considered species model with two stage-structures, but in real life many mammals (such as humans) growth is divided into three stages: childhood, adulthood, and old age; and almost all the insect growths are divided into eggs, larvae, and adults of three stages. Therefore, the three-stage- structure predator-prey model is studied that is more close to the reality. Recently, more and more papers, but still not too much, considered models with three-stage-structure such as [10–12].

Arditi and Ginzburg [13] first put forward the following ratio-dependent predator-prey system:

(1) $$\begin{array}{c} \dot{x}=x\left(a-bx\right)-\frac{cxy}{my+x},\\ \dot{y}=y\left(-d+\frac{fx}{my+x}\right). \end{array}$$

Later, Fan and Kuang [14] have explored the dynamics of the nonautonomous, spatially homogeneous, and continuous time predator-prey system with the Beddington-DeAngelis functional response in a more general form:

(2) $$\begin{array}{c} \dot{x}=x\left(a\left(t\right)-b\left(t\right)x\right)-\frac{c\left(t\right)xy}{\alpha \left(t\right)+\beta \left(t\right)x+\gamma \left(t\right)y},\\ \dot{y}=y\left(-d\left(t\right)+\frac{f\left(t\right)xy}{\alpha \left(t\right)+\beta \left(t\right)x+\gamma \left(t\right)y}\right). \end{array}$$

Recently, Chen et al. [8] studied a delayed predator-prey system with Holling II type response and stage-structure for predator of the form

(3) x˙(t)=x(t)(r(t)−a(t)x(t−τ1(t)))−b(t)x(t)y2(t)1+mx(t),y˙1(t)=c(t)x(t−τ2(t))y2(t−τ2(t))1+mx(t−τ2(t))−(D(t)+v1(t))y1(t),y˙2(t)=D(t)y1(t)−v2(t)y2(t).

In the present paper, motivated by the above work, we investigate a time delay predator-prey system with three-stage-structure for predator and Beddington-DeAngelis functional response of the form

(4) x˙1(t)=d(t)y(t−τ2(t))x2(t−τ2(t))α(t)+β(t)y(t−τ2(t))+γ(t)x2(t−τ2(t))−(d1(t)+r1(t))x1(t),x˙2(t)=r1(t)x1(t)−d2(t)x2(t)−r2(t)x2(t)−e2(t)x22(t),x˙3(t)=r2(t)x2(t)−d3(t)x3(t),y˙(t)=y(t)(b(t)−a(t)x(t−τ1(t))−c(t)x2(t)α(t)+β(t)y(t)+γ(t)x2(t)),

with initial conditions

(5) xi(θ)=φi(θ)>0 (i=1,2,3),y(θ)=φ4(θ)>0, for  θ∈[−τ,0],xi(0)>0, i=1,2,3,  y(0)>0,

where *x*_1_(*t*), *x*_2_(*t*), and *x*_3_(*t*) are the densities of immature, mature, and the old age predator at time *t*, respectively; *y*(*t*) represents the density of prey at time *t*. *τ* = max⁡_*t*∈[0,*ω*]_{*τ*_1_(*t*), *τ*_2_(*t*)}, *τ*_1_(*t*) ≥ 0 is delay due to prey densities and *τ*_2_(*t*) ≥ 0 is delay due to gestation of predator. The death rates of the immature, mature, and old age predator are proportional to the fact that existing immature, mature, and old age predator population with respective proportionality *d*_1_(*t*), *d*_2_(*t*), *d*_3_(*t*), *r*_1_(*t*) denote the rate of transformation from immature predator into mature predator; *r*_2_(*t*) is the rate of transformation from mature predator into old age predator; *k*(*t*) = (*d*(*t*)/*c*(*t*)) (0 < *k*(*t*) ≤ 1) is the transformation coefficient from prey into the immature predators. Let *d*_1_(*t*) + *r*_1_(*t*) = *m*(*t*) and *d*_2_(*t*) + *r*_2_(*t*) = *n*(*t*).

In this paper, we always make the following assumption for the system (4).

(H1) The coefficients *a*(*t*), *b*(*t*), *c*(*t*), *d*(*t*), *d*_*i*_(*t*) (*i* = 1,2, 3), *α*(*t*), *β*(*t*), *γ*(*t*), *e*_2_(*t*) and *r*_*i*_(*t*) (*i* = 1,2) are all positive *ω*-periodic continuous function in *R* = [0, *∞*).

The organization of this paper is as follows. In the next section, we give two lemmas for preparing the study of the existence of positive periodic solutions. In Section 3, by using Gains and Mawhin's continuation theorem of coincidence degree theory, a sufficient condition on the existence of positive periodic solutions for the model is obtained.

## 2. Preliminaries

For ecological reasons, we consider the initial function of the system (4) only in *R*_+_^4^ × *R*_+_^1^ = {(*x*_1_(*t*), *x*_2_(*t*), *x*_3_(*t*), *y*(*t*))^*T*^ : *x*_*i*_ ≥ 0,  *y* ≥ 0,  *i* = 1,2, 3}×[−*τ*, 0].

Supposing that *f*(*t*), *t* ∈ [0, *∞*) is a continuous function with periodic *ω*, we denote

(6) fL=min⁡t∈[0,ω]{f(t)},fM=max⁡t∈[0,ω]{f(t)},  f−=1ω∫0ωf(t)dt.

Using the above definitions, the coefficients of the system (4) not only are all positive and periodic function with common periodic solution but also satisfy the following two items and hold true:

(7) min⁡i=1,2,3{aL,bL,cL,dL,mL,nL,diL,riL,e2L,αL,βL,γL}>0,max⁡i=1,2,3{aM,bM,cM,dM,mM,nM,diM,riM,e2M,αM,βM,γM}  <+∞.

In order to obtain the existence of at least one periodic solution of the system (4), the method used the applications of the continuation theorem of coincidence degree. For convenience, we introduce a few concepts and results about the coincidence degree as follows.

Let *X*, *Y* be real Banach spaces, let *L* : Dom⁡*L* ⊂ *X* → *Z* be a linear mapping, and let *N* : *X* → *Y* be a continuous mapping. The mapping *L* will be called a Fredholm mapping of index zero if dim⁡⁡Ker⁡*L* = co⁡dim⁡⁡*Im*⁡*L* < *∞* and *Im*⁡*L* is closed in *Y*. If *L* a is Fredholm mapping of index zero, then there exist continuous projectors *P* : *X* → *X* and *Q* : *Z* → *Z* such that *Im*⁡*P* = Ker⁡*L* and Ker⁡*Q* = *Im*⁡*L* = *Im*⁡(*I* − *Q*). It follows that the restriction *L*|_Dom⁡*L*∩Ker⁡*P*_ : (*I* − *P*)*X* → *Im*⁡*L* is invertible. Denote the inverse of *L*|_Dom⁡*L*∩Ker⁡*P*_ by *K*_*P*_.

Let *Ω* be an open bounded subset of *X* and denote the closure of *Ω* by $\bar{\Omega }$. A mapping *N* is said to be *L*-compact on $\bar{\Omega }$. If $QN(\bar{\Omega })$ is bounded and $K_{P}(I-Q)N:\bar{\Omega }\to X$ is compact, since *Im*⁡*Q* is isomorphic to Ker*L*, then there exists an isomorphism *J* : *Im*⁡*Q* → Ker⁡*L*.

**Lemma 1 (Mawhin's continuation theorem [15, page 40])**

Let *X*, *Y* be two Banach spaces, let *L* be a Fredholm mapping of index zero and let $N:\bar{\Omega }\to Y$ be a continuous operator which is *L*-compact on $\bar{\Omega }$. Assume that,

(a) for each *λ* ∈ (0,1), *x* ∈ ∂*Ω*∩*Dom* *L*, *Lx* ≠ *λNx*;
(b) for each *x* ∈ ∂*Ω*∩*Ker* *L*, *QNx* ≠ 0;
(c) *deg* (*JQN*, *Ω*∩*Ker* *L*, 0) ≠ 0.

Then the operator equation *Lx* = *Nx* has at least one solution in $\bar{\Omega }\cap Dom\;L$.

**Lemma 2**

*in* *tR*_+_^4^ is a positively invariant region of the system (4).

## 3. Existence of Periodic Solution

**Theorem 3**

Assume that the system (4) satisfies (H1) and the following:

(H2) *r*_1_^*M*^ *d* ^*M*^ > *n* ^*L*^ *m* ^*L*^ *β* ^*L*^;
(H3) $\overset{-}{b}>\bar{\left(c/r\right)}$;
(H4) *r*_1_^*L*^ *d* ^*L*^ *A*_4_ > *m* ^*M*^ *n* ^*M*^(*α* ^*M*^ + *β* ^*M*^ *A*_4_).

Then the system (4) with initial condition (5) has at least one *ω*-periodic solution.

**Proof**

Let

(8) $$\begin{array}{c} x_{i}\left(t\right)=e^{u_{i}(t)}\;\left(i=1,2,3\right),\;\;y\left(t\right)=e^{u_{4}(t)}. \end{array}$$

Then the system (4) transforms correspondingly to the following equations:

(9) u˙1(t)=d(t)eu4(t−τ2(t))eu2(t−τ2(t))−u1(t)α(t)+β(t)eu4(t−τ2(t))+γ(t)eu2(t−τ2(t))−m(t),u˙2(t)=r1(t)eu1(t)−u2(t)−n(t)−e2(t)eu2(t),u˙3(t)=r2(t)eu2(t)−u3(t)−d3(t),u˙4(t)=b(t)−a(t)eu4(t−τ1(t))−c(t)eu2(t)α(t)+β(t)eu4(t)+γ(t)eu2(t).

It is easy to see that if the system (9) has a *ω*-periodic solution u*(*t*) = (*u*_1_*, *u*_2_*, *u*_3_*, *u*_4_*)^*T*^, then the system (4) has correspondingly a positive *ω*-periodic solution (*x*_1_*, *x*_2_*, *x*_3_*, y*)^*T*^ = (*e* ^*u*_1_*^, *e* ^*u*_2_*^, *e* ^*u*_3_*^, *e* ^*u*_4_*^)^*T*^. Thus, to complete the proof, suffice it to say that the system (9) has at least one positive *ω*-periodic solution.

Define *X* = *Y* = {*u*(*t*) = (*u*_1_(*t*), *u*_2_(*t*), *u*_3_(*t*), *u*_4_(*t*))^*T*^ ∈ *C*(*R*, *R* ^4^), *u*(*t* + *ω*) = *u*(*t*)}, and ||*u*|| = ∑_*i*=1_^4^max⁡_*t*∈[0,*ω*]_ | *u*_*i*_(*t*)|; then *X* and *Y* are Banach spaces with the norm ||·||.

Let *Nu* = (*N*_1_(*t*), *N*_2_(*t*), *N*_3_(*t*), *N*_4_(*t*))^*T*^, *u* ∈ *X*, $Lu=\dot{u}(t)=du(t)/dt$, *Pu* = (1/*ω*)∫_0_^*ω*^ *u*(*t*)*dt*, *u* ∈ *X*, and *Qy* = (1/*ω*)∫_0_^*ω*^ *y*(*t*)*dt*, *y* ∈ *Y*, where

(10) N1(t)=d(t)eu4(t−τ2(t))eu2(t−τ2(t))−u1(t)α(t)+β(t)eu4(t−τ2(t))+γ(t)eu2(t−τ2(t))−m(t),N2(t)=r1(t)eu1(t)−u2(t)−n(t)−e2(t)eu2(t),N3(t)=r2(t)eu2(t)−u3(t)−d3(t),N4(t)=b(t)−a(t)eu4(t−τ1(t))−c(t)eu2(t)α(t)+β(t)eu4(t)+γ(t)eu2(t).

Thus, the system (9) can be written in the form *Lu* = *Nu*,  *u* ∈ *X*.

Obviously, Ker⁡*L* = *R* ^4^, *Im*⁡*L* = {*u*(*t*) ∈ *Z*, ∫_0_^*ω*^ *u*(*t*)*dt* = 0} is closed in *Y* and dim⁡⁡Ker⁡*L* = 4 = Codim⁡⁡*Im*⁡*L* and *P*, *Q* are continuous projectors such that *Im*⁡*P* = Ker⁡*L*, Ker⁡*Q* = *Im*⁡*L* = *Im*⁡(*I* − *Q*). Therefore, *L* is a Fredholm mapping of index zero. Furthermore, the inverse *K*_*P*_ : *Im*⁡*L* → Ket *P*∩Dom⁡*L* exists and has the form

(11) $$\begin{array}{c} K_{P}\left(u\right)=\overset{t}{\underset{0}{\int }}u\left(s\right)ds-\frac{1}{\omega }\overset{\omega }{\underset{0}{\int }}\overset{t}{\underset{0}{\int }}u\left(s\right)ds\;dt. \end{array}$$

Thus

(12) QNu=1ω∫0ωN(t)dt,KP(I−Q)Nu=∫0tN(s)ds−1ω∫0ω∫0tNu(s)ds dt−(tω−12)∫0ωNu(s)ds.

Obviously, *QN* and *K*_*P*_(*I* − *Q*)*N* are continuous by the Lebesgue theorem; it is not difficult to show that $QN(\bar{\Omega })$ is bounded and $\bar{K_{P}(I-Q)N(\bar{\Omega })}$ is compact for any open bounded set *Ω* ⊂ *X* by the Arzela-Ascoli theorem. Hence *N* is *L*-compact on $\bar{\Omega }$ for any open bounded set *Ω* ⊂ *X*.

In order to apply Lemma 1, we need to search for an appropriate open and bounded subset *Ω* ⊂ *X*. Corresponding to the operator equation, *Lu* = *λ* *Nu*, *λ* ∈ (0,1), we have

(13) u˙1(t)=λ(d(t)eu4(t−τ2(t))eu2(t−τ2(t))−u1(t)α(t)+β(t)eu4(t−τ2(t))+γ(t)eu2(t−τ2(t))−m(t)),u˙2(t)=λ(r1(t)eu1(t)−u2(t)−n(t)−e2(t)eu2(t)),u˙3(t)=λ(r2(t)eu2(t)−u3(t)−d3(t)),u˙4(t)=λ(b(t)−a(t)eu4(t−τ1(t))−c(t)eu2(t)α(t)+β(t)eu4(t)+γ(t)eu2(t)).

Suppose that *u* = *u*(*t*) ∈ *X* is a solution to (13) for a certain *λ* ∈ (0,1). Integrating (13) over the interval [0, *ω*] leads to

(14) $$\begin{array}{c} \overset{\omega }{\underset{0}{\int }}\frac{d\left(t\right)e^{u_{4}(t-\tau_{2}(t))}e^{u_{2}(t-\tau_{2}(t))-u_{1}(t)}}{\alpha \left(t\right)+\beta \left(t\right)e^{u_{4}(t-\tau_{2}(t))}+\gamma \left(t\right)e^{u_{2}(t-\tau_{2}(t))}}dt=\overset{-}{m}\omega , \end{array}$$

(15) $$\begin{array}{c} \overset{\omega }{\underset{0}{\int }}r_{1}\left(t\right)e^{u_{1}(t)-u_{2}(t)}dt=\overset{-}{n}\omega +\overset{\omega }{\underset{0}{\int }}e_{2}\left(t\right)e^{u_{2}(t)}dt, \end{array}$$

(16) $$\begin{array}{c} \overset{\omega }{\underset{0}{\int }}r_{2}\left(t\right)e^{u_{2}(t)-u_{3}(t)}dt={\overset{-}{d}}_{3}\omega , \end{array}$$

(17) ∫0ω[a(t)eu4(t−τ1(t))+c(t)eu2(t)α(t)+β(t)eu4(t)+γ(t)eu2(t)]dt  =b−ω.

It follows from (14) to (17) that

(18) ∫0ω|u˙1(t)|dt  =λ∫0ω|d(t)eu4(t−τ2(t))eu2(t−τ2(t))−u1(t)α(t)+β(t)eu4(t−τ2(t))+γ(t)eu2(t−τ2(t))   −m(t)|dt≤2m−ω=:l1,

(19) ∫0ω|u˙2(t)|dt  =λ∫0ω|r1(t)eu1(t)−u2(t)−n(t)−e2(t)eu2(t)|dt  ≤2n−ω+2∫0ωe2(t)eu2(t)dt,

(20) ∫0ω|u˙3(t)|dt  =λ∫0ω|r2(t)eu2(t)−u3(t)−d3(t)|dt  ≤2d−3ω=:l3,

(21) ∫0ω|u˙4(t)|dt  =λ∫0ω|b(t)−a(t)eu4(t−τ1(t))   −c(t)eu2(t)α(t)+β(t)eu4(t)+γ(t)eu2(t)|dt  ≤2b−ω=:l4.

Multiplying the first equation of (13) by *e* ^*u*_1_(*t*)^ and integrating it over [0, *ω*] lead to

(22) ∫0ωm(t)eu1(t)dt  =∫0ωd(t)eu4(t−τ2(t))eu2(t−τ2(t))α(t)+β(t)eu4(t−τ2(t))+γ(t)eu2(t−τ2(t))dt,

which implies

(23) mL∫0ωeu1(t)dt≤∫0ωd(t)β(t)eu2(t−τ2(t))dt≤dMβL∫0ωeu2(t−τ2(t))dt,∫0ωeu2(t)dt=∫τ2(s)ω−τ2(s)eu2(t)dt=∫0ωeu2(t−τ2(t))dt≥mLβLdM∫0ωeu1(t)dt.

Similarly, multiplying the second equation of (13) by *e* ^*u*_2_(*t*)^ and integrating it over [0, *ω*] give

(24) $$\begin{array}{c} \overset{\omega }{\underset{0}{\int }}e_{2}\left(t\right)e^{2u_{2}(t)}dt+\overset{\omega }{\underset{0}{\int }}n\left(t\right)e^{u_{2}(t)}dt=\overset{\omega }{\underset{0}{\int }}r_{1}\left(t\right)e^{u_{1}(t)}dt \end{array}$$

which yields

(25) $$\begin{array}{c} e_{2}^{L}\overset{\omega }{\underset{0}{\int }}e^{2u_{2}(t)}dt+n^{L}\overset{\omega }{\underset{0}{\int }}e^{u_{2}(t)}dt\leq r_{1}^{M}\overset{\omega }{\underset{0}{\int }}e^{u_{1}(t)}dt. \end{array}$$

By using the inequalities (∫_0_^*ω*^ *e* ^*u*_2_(*t*)^ *dt*)^2^ ≤ *ω*∫_0_^*ω*^ *e* ^2*u*_2_(*t*)^ *dt* and from (23) and (25) as well as (H1), we have

(26) ∫0ωeu2(t)dt≥mLβLdM∫0ωeu1(t)dt≥mLβLdMr1M(e2L∫0ωe2u2(t)dt+nL∫0ωeu2(t)dt)≥mLβLdMr1M[e2Lω(∫0ωeu2(t)dt)2+nL∫0ωeu2(t)dt].

Hence

(27) $$\begin{array}{c} \overset{\omega }{\underset{0}{\int }}e^{u_{2}(t)}dt\leq \omega \frac{d^{M}r_{1}^{M}-n^{L}m^{L}\beta^{L}}{m^{L}\beta^{L}e_{2}^{L}}=:\omega B_{2}. \end{array}$$

From (23) and (27), we have

(28) $$\begin{array}{c} \overset{\omega }{\underset{0}{\int }}e^{u_{1}(t)}dt\leq \frac{d^{M}}{m^{L}\beta^{L}}\overset{\omega }{\underset{0}{\int }}e^{u_{2}(t)}dt\leq \omega \frac{B_{2}d^{M}}{m^{L}\beta^{L}}=:\omega B_{1}. \end{array}$$

Similarly, multiplying the third equation of (13) by *e* ^*u*_3_(*t*)^ and integrating it over [0, *ω*] lead to

(29) $$\begin{array}{c} \overset{\omega }{\underset{0}{\int }}d_{3}\left(t\right)e^{u_{3}(t)}dt=\overset{\omega }{\underset{0}{\int }}r_{2}\left(t\right)e^{u_{2}(t)}dt. \end{array}$$

And, together with (27), we obtain

(30) $$\begin{array}{c} \overset{\omega }{\underset{0}{\int }}e^{u_{3}(t)}dt\leq \frac{r_{2}^{M}}{d_{3}^{L}}\overset{\omega }{\underset{0}{\int }}e^{u_{2}(t)}dt\leq \omega \frac{B_{2}r_{2}^{M}}{d_{3}^{L}}=:\omega B_{3}. \end{array}$$

Integrating the fourth equation of (13) over [0, *ω*] leads to

(31) ∫0ωa(t)eu4(t−τ1(t))dt  =∫0ωb(t)dt−∫0ωc(t)eu2(t)α(t)+β(t)eu4(t)+γ(t)eu2(t)dt,

which implies

(32) $$\begin{array}{c} \overset{\omega }{\underset{0}{\int }}e^{u_{4}(t)}dt\leq \omega \frac{b^{M}}{a^{L}}=:\omega B_{4}. \end{array}$$

Since *u*(*t*) ∈ *X*, there exist *ξ*_*i*_, *η*_*i*_ ∈ [0, *ω*] such that

(33) $$\begin{array}{c} u_{i}\left(\xi_{i}\right)=\underset{t\in \left[0,\omega \right]}{\max}u_{i}\left(t\right),\;u_{i}\left(\eta_{i}\right)=\underset{t\in \left[0,\omega \right]}{\min}u_{i}\left(t\right),\;i=1,2,3, \end{array}$$

in which, together with (27), (28), (30), and (32), we deduce

(34) $$\begin{array}{c} u_{i}\left(\xi_{i}\right)\leq \ln B_{i},\;i=1,2,3,4. \end{array}$$

In view of (19) and (27), we have

(35) $$\begin{array}{c} \overset{\omega }{\underset{0}{\int }}\left|{\dot{u}}_{2}\left(t\right)\right|dt\leq 2\overset{-}{n}\omega +2\omega e_{2}^{M}B_{2}=:l_{2}. \end{array}$$

Keeping in mind (18), (20), (21), and (34), we obtain that for *t* ∈ [0, *ω*]

(36) $$\begin{array}{c} u_{i}\left(t\right)\leq u_{i}\left(\xi_{i}\right)+\overset{\omega }{\underset{0}{\int }}\left|{\dot{u}}_{i}\left(t\right)\right|dt\leq \ln B_{i}+l_{i},\;i=1,2,3,4. \end{array}$$

From (17), (33), and (H3), we have

(37) aMωeu4(η4)≥aM∫0ωeu4(t)dt=aM∫0ωeu4(t−τ1(t))dt=∫0ωb(t)dt −∫0ωc(t)eu2(t)α(t)+β(t)eu4(t)+γ(t)eu2(t)dt≥b−ω−(cγ)¯ω.

Set $A_{4}=\left(\overset{-}{b}-\bar{\left(c/\gamma \right)}\right)/a^{M}$, and hence

(38) $$\begin{array}{c} e^{u_{4}(\eta_{4})}\geq A_{4}. \end{array}$$

Equation (33) leads to

(39) d(η1)eu4(η1−τ2(η1))eu2(η1−τ2(η1))−u1(η1)α(η1)+β(η1)eu4(η1−τ2(η1))+γ(η1)eu2(η1−τ2(η1))  −m(η1)=0,r1(η2)eu1(η2)−u2(η2)−n(η2)−e2(η2)eu2(η2)=0,r2(η3)eu2(η3)−u3(η3)−d3(η3)=0,b(η4)−a(η4)eu4(η4−τ1(η4))  −c(η4)eu2(η4)α(η4)+β(η4)eu4(η4)+γ(η4)eu2(η4)=0.

Taking into account (38) and the monotonicity of *g*(*x*) = *x*/(*x* + *l*) leads to

(40) mMeu1(η1)≥m(η1)eu1(η1)=d(η1)eu4(η1−τ2(η1))eu2(η1−τ2(η1))−u1(η1)α(η1)+β(η1)eu4(η1−τ2(η1))+γ(η1)eu2(η1−τ2(η1))≥dLA4eu2(η2)αM+βMA4+γMeu2(η2),

(41) e2Me2u2(η2)+nMeu2(η2)  ≥e2(η2)e2u2(η2)+n(η2)eu2(η2)  =r1(η2)eu1(η2)≥r1Leu1(η1),

(42) d3Meu3(η3)≥d3(η3)eu3(η3)=r2(η3)eu2(η3)≥r2Leu2(η2).

It follows from (40) and (41) that

(43) $$\begin{array}{c} e_{2}^{M}e^{u_{2}(\eta_{2})}+n^{M}\geq \frac{r_{1}^{L}d^{L}A_{4}}{m^{M}\left(\alpha^{M}+\beta^{M}A_{4}+\gamma^{M}e^{u_{2}(\eta_{2})}\right)}, \end{array}$$

which yields

(44) eu2(η2)≥(−(e2M(αM+βMA4)+nMγM)    +(e2M(αM+βMA4)+nMγM)2+4A5)    ×(2e2MγM)−1=:A2,

where *A*_5_ = *e*_2_^*M*^ *γ* ^*M*^((*r*_1_^*L*^ *d* ^*L*^ *A*_4_/*m* ^*M*^) − *n* ^*M*^(*α* ^*M*^ + *β* ^*M*^ *A*_4_)). From (40), (42), and (44), we have

(45) $$\begin{array}{c} e^{u_{1}(\eta_{1})}\geq \frac{d^{L}A_{4}A_{2}}{m^{M}\left(\alpha^{M}+\beta^{M}A_{4}+\gamma^{M}A_{2}\right)}=:A_{1},\\ e^{u_{3}(\eta_{3})}\geq \frac{r_{2}^{L}A_{2}}{d_{3}^{M}}=:A_{3}, \end{array}$$

which, together with (38), gives

(46) $$\begin{array}{c} u_{i}\left(\eta_{i}\right)\geq \ln A_{i},\;i=1,2,3,4. \end{array}$$

Moreover, from (18) to (21) and (35), we obtain

(47) ui(t)≥ui(ηi)−∫0ω|u˙i(t)|dt≥ln⁡⁡Ai−li, t∈[0,ω],  i=1,2,3,4.

It follows from (36) and (47) that

(48) max⁡t∈[0,ω]⁡|ui(t)|<max⁡⁡{|ln⁡Bi+li|,|ln⁡⁡Ai−li|}=Ri,i=1,2,3,4.

Clearly,  *R*_*i*_ (*i* = 1,2, 3,4) is independent of *λ*. From condition of Theorem 3, it is easy to know that there exist points *ζ*_*i*_ ∈ [0, *ω*], *i* = 1,2, such that the algebraic equations,

(49) $$\begin{array}{c} \frac{\overset{-}{d}e^{u_{4}}e^{u_{2}-u_{1}}}{\alpha \left(\zeta_{1}\right)+\beta \left(\zeta_{1}\right)e^{u_{4}}+\gamma \left(\zeta_{1}\right)e^{u_{2}}}-\bar{m}=0,\\ \bar{r_{1}}e^{u_{1}-u_{2}}-\overset{-}{n}-\bar{e_{2}}e^{u_{2}}=0,\\ \bar{r_{2}}e^{u_{2}-u_{3}}-\bar{d_{3}}=0,\\ \overset{-}{b}-\overset{-}{a}e^{u_{4}}-\frac{\overset{-}{c}e^{u_{2}}}{\alpha \left(\zeta_{2}\right)+\beta \left(\zeta_{2}\right)e^{u_{4}}+\gamma \left(\zeta_{2}\right)e^{u_{2}}}=0, \end{array}$$

have a unique solution (*v*_1_*, *v*_2_*, *v*_3_*, *v*_4_*)^*T*^.

Set *H* = ∑_*i*=1_^4^ *R*_*i*_ + *R*_0_, where *R*_0_ is taken sufficiently large such that the unique solution to (49) satisfies ||(*v*_1_*, *v*_2_*, *v*_3_*, *v*_4_*)^*T*^|| = |*v*_1_* | +|*v*_2_* | +|*v*_3_* | +|*v*_4_* | <*R*_0_.

We take *Ω* = {*u*(*t*) = (*u*_1_(*t*), *u*_2_(*t*), *u*_3_(*t*), *u*_3_(*t*))^*T*^ ∈ *X* : ||*u*|| < *M*}; then *Ω* satisfies the condition (a) of Lemma 1. When *u* ∈ ∂*Ω*∩*ker*⁡⁡*L* = ∂*Ω*∩*R* ^4^, *u* is a constant vector in *R* ^4^ with ||*u*|| = *H*; then we have

(50) $$\begin{array}{c} QNu=\left[\begin{array}{c} \frac{\overset{-}{d}e^{u_{4}}e^{u_{2}-u_{1}}}{\alpha \left(\zeta_{1}\right)+\beta \left(\zeta_{1}\right)e^{u_{4}}+\gamma \left(\zeta_{1}\right)e^{u_{2}}}-\bar{m}\\ \bar{r_{1}}e^{u_{1}-u_{2}}-\overset{-}{n}-\bar{e_{2}}e^{u_{2}}\\ \bar{r_{2}}e^{u_{2}-u_{3}}-\bar{d_{3}}\\ \overset{-}{b}-\overset{-}{a}e^{u_{4}}-\frac{\overset{-}{c}e^{u_{2}}}{\alpha \left(\zeta_{2}\right)+\beta \left(\zeta_{2}\right)e^{u_{4}}+\gamma \left(\zeta_{2}\right)e^{u_{2}}} \end{array}\right]=\left[\begin{array}{c} 0\\ 0\\ 0\\ 0 \end{array}\right]. \end{array}$$

This proves that condition (b) of Lemma 1 is satisfied.

We define the homotopy *J* : Dom⁡*L* × [0,1] → *X*, and a direct calculation shows that

(51) deg⁡⁡(JQNu,Ω∩Ker⁡L,(0,0,0,0)T)  =sgn⁡(a¯d¯r1¯r2¯e2u4+u2−u3(α(ζ1)+β(ζ1)eu4+γ(ζ1)eu2)2)=1≠0.

Therefore *Ω* satisfies all the conditions of Lemma 1. Thus, by Lemma 1, we conclude that *Lu* = *Nu* has at least one solution in $\mathrm{Dom}L\cap \overset{-}{\Omega }$; that is, the system (9) has at least one *ω*-periodic solution in *Ω*. Then, there exists at least one *ω*-periodic solution for the system (4). The proof is now completed.

## 4. Discussion

In this paper, we have considered a predator-prey system with three-stage-structure for predator, time delay due to predator gestation and prey densities, and Beddington-DeAngelis functional response. We have derived criteria for the existence of a positive periodic solution. The method used to obtain the main result involves application of Mawhin's continuous theorem of coincidence degree theory and integral inequalities.

Chen et al. [8] considered a delayed predator-prey system with Holling II type response and structure for predator. If the predator *x*_2_ is absent and the coefficients *α*(*t*) ≡ 1, *β*(*t*) ≡ *m*(constant), and *γ*(*t*) ≡ 0, the corresponding subsystems of the system (4) are system (3); that is, the system of [8] belongs to the case of the system (4). The conditions for the existence of a positive periodic solution of the system (4) remain the same if delay is absent; thus the delay is “harmless” for the existence of a positive periodic solution of the system (4).

In a word, despite considering the three-stage-structure, people can more protect the beneficial insect from dying out and under the preconditions; the actualization of the periodic changes is more convenient and realistic. The results accord with the change in the natural environments, so the consideration of stage-structure is necessary and important.
